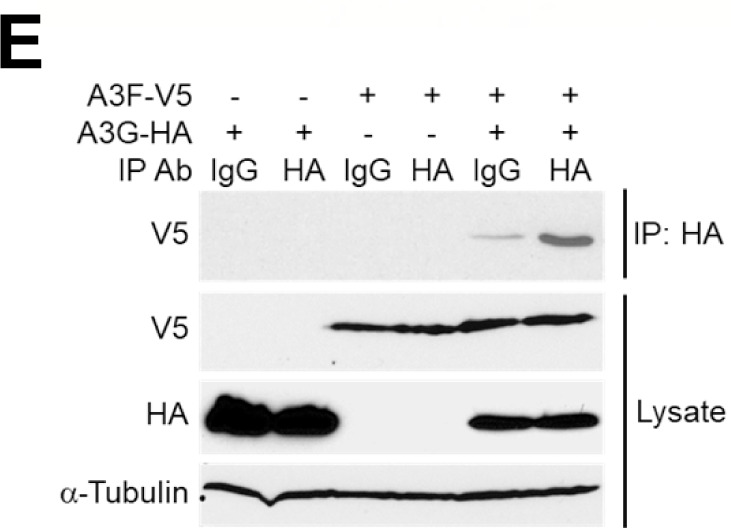# Erratum for Ara et al., “Mechanism of Enhanced HIV Restriction by Virion Coencapsidated Cytidine Deaminases APOBEC3F and APOBEC3G”

**DOI:** 10.1128/jvi.02204-24

**Published:** 2025-01-16

**Authors:** Anjuman Ara, Robin P. Love, Tyson B. Follack, Khawaja A. Ahmed, Madison B. Adolph, Linda Chelico

## ERRATUM

Volume 91, no. 3, e02230-16, 2017, https://doi.org/10.1128/jvi.02230-16. Page 4: Figure 1E should appear as shown in this erratum. In the label above the blot, one lane was labeled “V5” but should have been labeled “HA.”

**Fig 1:**